# The Kinematic Control During the Backward Gait and Knee Proprioception: Insights from Lesions of the Anterior Cruciate Ligament

**DOI:** 10.2478/hukin-2014-0032

**Published:** 2014-07-08

**Authors:** Davide Viggiano, Katia Corona, Simone Cerciello, Michele Vasso, Alfredo Schiavone-Panni

**Affiliations:** 1 Dept. Medicine and Health Sciences, University of Molise, Campobasso, Italy.

**Keywords:** backward walking, treadmill, knee joint, step length, ligament reconstruction

## Abstract

An already existing large volume of work on kinematics documents a reduction of step length during unusual gaits, such as backward walking. This is mainly explained in terms of modifications of some biomechanical properties. In the present study, we propose that the proprioceptive information from the knee may be involved in this change of motor strategy. Specifically, we show that a non-automated condition such as backward walking can elicit different motor strategies in subjects with reduced proprioceptive feedback after anterior cruciate ligament lesion (ACL). For this purpose, the kinematic parameters during forward and backward walking in subjects with ACL deficit were compared to two control groups: a group with intact ACL and a group with surgically reconstructed ACL. The knee proprioception was tested measuring the threshold for detection of passive knee motion. Subjects were asked to walk on a level treadmill at five different velocities (1–5km/h) in forward and backward direction, thereby calculating the cadence and step length. Results showed that forward walking parameters were largely unaffected in subjects with ACL damage. However, they failed to reduce step length during backward walking, a correction that was normally observed in all control subjects and in subjects with normal proprioceptive feedback after ACL reconstruction. The main result of the present study is that knee proprioception is an important signal used by the brain to reduce step length during the backward gait. This can have a significant impact on clinical evaluation and rehabilitation.

## Introduction

The kinematic control of walking has been the object of a large number of studies. Multiple brain regions appear to be involved in this complex task, from spinal circuits (central pattern generators) to cerebellar (Morton and Bastian, 2006), striatal and cortical ones (Elble et al., 1996). One of the major challenges is the exact understanding of how the brain modifies the walking pattern to face instable surfaces: which type of sensory feedback is used and how this interacts with the biomechanical system?

Although energetically inefficient, step shortening is a well-known strategy adopted by the brain in presence of uncertainty or instability. For instance, it is used during walking with obstacle avoidance in elderly subjects (Chen et al., 1991; Weerdesteyn et al., 2005). Walking backwards is a non-automated situation, particularly when executed on a treadmill, characterized by a smaller step size and increased step-to-step variability, without modifications of the cadence (Laufer, 2005). The main hypothesis for this compensatory adjustment has considered the modifications in biomechanical properties of lower limbs. However, backward walking on a treadmill challenges the stability and thus it is possible that step shortening can be partially attributed to the subjects’ uncertainty and attempt to maintain stability. If this is the case, proprioceptive information from lower limbs may be important for proper correction of step length.

The anterior cruciate ligament (ACL) lesion is a common sports injury and accounts for more than 50% of all ligament injuries (Bollen, 1998; Prodromos et al., 2007; Gianotti et al., 2009). The ACL plays an important role in knee biomechanical stabilization; however, it is also a source of proprioceptive information in static and dynamic situations, such as walking. Therefore, the ACL lesion leads not only to biomechanical modifications, but also to proprioceptive deficits (Denti et al., 1994; Bonfim et al., 2003). These impairments are accompanied by minor deficits during normal walking such as subjective knee instability (Eastlack et al., 1999), poor muscular control (Tsepis et al., 2004) and minor knee biomechanical changes (Ferber, 2001). A recent report also showed loss of optimal step-to-step variability in backward walking (BW) in ACL deficient (Zampeli et al., 2010). It is unclear whether these modifications reflect the mechanical impairment or the proprioceptive loss.

The presence of only minor changes in the gait pattern after the ACL lesion might be due to the high degree of automation of this motor sequence. Therefore, it is possible that proprioceptive modifications after ACL damage become more evident during unusual movements, such as during BW. The hypothesis of the present study is that some of the kinematic adjustments occurring during BW require proprioceptive feedback from lower limbs and, therefore, are modified in presence of proprioceptive loss after ACL damage. For this purpose, the kinematic modifications during BW in subjects with ACL deficiency (ACL-D) were analysed. These subjects were compared to two control groups: a group with an intact ACL (ACL-I) and a group with a surgically reconstructed ACL (ACL-R).

## Material and Methods

The study group consisted of 15 male ACL deficient (ACL-D) subjects (age 30±4.8 yr, body height 174.6±4.86 cm, body mass 77.6±6.8 kg, BMI 25.7±2.0; data reported as mean±SD), 15 subjects who had undergone ACL reconstruction (age 24±4.6 yr, body height 179.6±5.7 cm, body mass 76.7±6.2 kg, BMI 23.8±1.71) and 15 subjects with intact ACL (ACL-I) (age 25±3.8 yr, body height 175.8±6.0 cm, body mass 73.4±6.1 kg, BMI 23.8±1.55). All subjects were male professional soccer players.

Exclusion criteria for the present study were: history of vestibular dysfunction, uncorrected vision problems, history of orthopaedic lower extremity injury and history of neurological disease or injury. All subjects with ACL damage or reconstruction were recruited at beyond 9 months after the injury or the surgery. None of the operated subjects referred swelling or pain or limiting gait deviation at the time of control.

Informed consent, as approved by Ethical Committee of the University of Molise, was obtained prior to testing. ACL deficiency was confirmed by clinical examination and MRI. Each subject completed a questionnaire regarding his medical history and physical activity level. Specifically, the questionnaire required the subjects to report the number of hours they devoted to sport activities during a week.

For ACL deficient subjects, incidence of giving way and ability to return to cutting and pivoting sport activities was recorded. All subjects were also interviewed using the Tegner & Lysholm and the IKDC scales.

### Knee proprioception testing

Conscious knee proprioception was tested measuring the threshold for detection of passive knee motion, according to a protocol modified by Barrack et al. (1984) and Courtney et al. (2005). Subjects were tested in a seated position, with the trunk at 60° inclination to encourage relaxation, with the knee flexed at 90° and the ankle in a neutral position. They were asked to close the eyes to prevent visual feedback. An inflated air splint was placed below the thigh. The foot was placed on a pedal attached to a custom system of pulleys. Specifically, we modified a spin cycle (Schwinn, Johnny G Pro Spin Bike; crank length: 17cm), stably connecting the foot of the subjects to the pedal so that large movements of the fly-wheel corresponded to very small movements of the pedal. The fly-wheel was connected to a linear encoder (Ergospeed, Salvabyte, Italy) which measured the tangential velocity of the fly-wheel. Since the movement of the fly-wheel was much larger than the movemnt of the pedal, the final movement precision that could be measured with the linear encoder was of 0.02mm.

Passive, slow (about 0.5°/sec) flexion and extension movements of the knee were accomplished through the fine movement of the fly-wheel. Blindfolded subjects were instructed to indicate when they detected movement or change in the position of the knee. For each limb a minimum of three trials in each direction (flexion-extension) was made. The displacement of the pedal (X) was then used to calculate the threshold to detection (Y, in degrees) using the following formula :

Y=arctan (X/R)

Where R= shank leg (medial joint line of the knee to inferior aspect of the medial malleolus).

### Treadmill walking

Subjects were asked to walk on a level treadmill (Run XT 500, TechnoGym, Italy) at five different velocities, from 1 to 5km/h, increasing the velocity in steps of 1km/h. Later, subjects were asked to walk backward on the same apparatus at the same speeds. White reflective markers (2 cm diameter) were attached to bone landmarks and body segments of the subject to track body movements; these were placed on: (i) the lateral ankle (along the flexion/extension axis of rotation at lateral malleolus), (ii) the lateral side of the knee joint (along the flexion/extension axis of rotation at lateral femoral condyle). The marker set used in this study was more simple than configurations commonly used in gait analyses such as the Helen Hayes marker set because the main interest was to test basic kinematic measurements (step length). Moreover, we used two additional markers, which are not included in the Hayes’ marker set: (i) the head of the fifth metatarsal, and (ii) the greater trochanter.

A high definition digital camera (DCR-TRV14E with Zeiss optics, Sony) was placed laterally and recorded the subject’s gait. Images were off line analysed using Kinovea software to calculate the cadence (number of steps per minute) and step length. Each subject walked for one minute at each velocity (1 to 5 km/h in steps of 1km/h) on the treadmill; the subsequent analysis of step length of each subject was then performed on 40 step cycles per each velocity step.

Since the adjustments of step length during backward walking depend upon the novelty of this gait sequence, subjects performed the backward gait sequence only once. However, subjects could familiarize with the tapis roulant in forward direction for one minute at the lowest speed (1 km/h), before recording of the gait started.

The gait cycle was recognized using Heel Strike and Toe Off in FW and Toe Contact and Heel Off in BW.

### Statistics

Differences for the threshold for passive motion detection and step length at various velocities were tested using multivariate ANOVA for repeated measures. Normality tests had been done before ANOVA testing. Multiple post-hoc comparisons were conducted using the LSD test to identify differences among groups. Multiple linear regression analysis was used to test correlation between the proprioception level, physical activity level and step length. Rejection threshold for null hypothesis was set at p≤0.05. Data are presented as mean and standard error.

## Results

Differences for the threshold for passive motion detection and step length at various velocities were tested using multivariate ANOVA for repeated measures. Normality tests had been done before ANOVA testing. Multiple post-hoc comparisons were conducted using the LSD test to identify differences among groups. Multiple linear regression analysis was used to test correlation between the proprioception level, physical activity level and step length. Rejection threshold for null hypothesis was set at p≤0.05. Data are presented as mean and standard error.

### Results

The group with lesioned ACL showed significantly lower scores in the Tegner & Lysholm and the IKDC scales compared to both the control group and the group with a reconstructed ACL ligament, as reported in Table 1.

**Figure 1:**
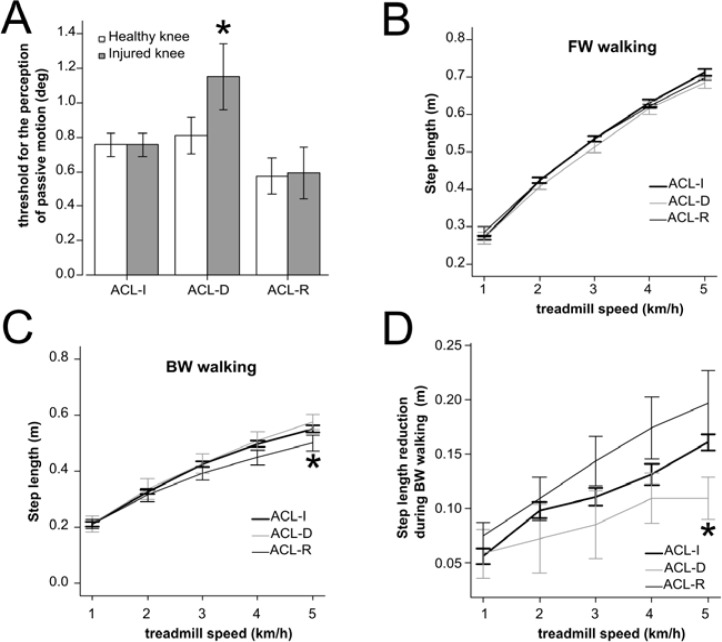
Knee proprioception and gait parameters during forward and backward walking in anterior cruciate ligament (ACL)- deficient subjects A: Subjects with ACL deficiency show a reduction in knee conscious proprioception measured as a threshold for the perception of passive motion. The healthy knee shows normal sensibility, comparable to that of control subjects and subjects with a reconstructed ACL. B: step length during treadmill walking at different speeds. C: step length during treadmill walking in backward direction, at different speeds. D: reduction of step length during backward walking at different speed. Subjects with ACL damage show smaller correction of step length compared to controls. ACL-I: intact ACL. ACL-D: damaged ACL; ACL-R: reconstructed ACL. Asterisk: p<0.05 vs controls.

Figure 1A shows that subjects with ACL damage (ACL-D) have reduced conscious proprioception in the injured knee, with normal sensibility in the intact knee, when compared to subjects with an intact ACL (ACL-I) (injured knee F=4.30, p=0.031; LSD test p=0.042 ACL-I vs. ACL-D; intact knee: F=1.733, p=0.20). Conversely, subjects with a reconstructed ACL (ACL-R) showed no significant differences in knee proprioception compared to the ACL-I (LSD test p=0.344 ACL-I vs. ACL-R).

Figure 1B shows that the three groups did not differ in their step length during forward walking on a treadmill at different speeds. In fact, repeated measures ANOVA showed no significant differences between the groups at different treadmill speed (F=0.621 p=0.54), without interaction effect (F=0.989, p=0.455).

Figure 1C shows that on average all subjects reduced their step length when performing BW on the treadmill compared to FW (Figures 1B and 1C). Moreover, during BW, ACL-R subjects decreased step length at higher treadmill speed. In fact, repeated measures ANOVA showed no significant group effect (F=1.20, p=0.32), but significant group per treadmill velocity interaction effect (F=3.43, p=0.002), which was due to the significant differences at high speed (LSD test p=0.047 ACL-I vs. ACL-R at treadmill speed of 5km/h) which were absent at low treadmill speed (LSD test p=0.640 ACL-I vs. ACL-R at a treadmill speed of 1km/h).

Figure 1D shows the ACL-D group differs in the correction of step length during BW from the other two groups: they fail to reduce the step length during BW (ANOVA test for repeated measures: group F=3.60, p=0.053; interaction factor (group × treadmill velocity): F=1.986, p=0.55; LSD test p=0.05 ACL-I vs. ACL-D, p=0.006 ACL-R vs. ACL-D at 5km/h); conversely, although on average ACL-R subjects showed a trend towards hypercorrection of step length (greater reduction of step length in BW), this difference did not reach statistical significance (p=0.12 ACL-R vs. ACL-I at 5km/h).

Regression analysis showed no significant correlation between step length correction (the difference in step length between the forward and backward gait) and physical activity (hours of exercise per week) (R=0.052, p = 0.756 at 2km/h, R=0.152, p = 0.364 at 3km/h). Conversely, a significant linear correlation was found between step length correction and proprioceptive sensibility of the knee (R=0.449, p=0.011 at 2km/h, R=0.329, p=0.07 at 3km/h).

## Discussion

The main result of the present study was that patients with poor proprioception of the knee after the ACL lesion did not adopt the step shortening strategy during backward walking. Conversely, the reconstruction of the ACL not only restored the conscious proprioception from the knee, but was accompanied by a possible hyper-correction of step length, with smaller step size during BW.

Backward walking is an interesting paradigm to understand gait control because it requires cyclic movements as in forward walking, but it is unusual, particularly on a treadmill. It has received great attention in the literature (Grasso et al., 1998; Nadeau et al., 2003; Choi and Bastian, 2007; Soda et al., 2013). The compensatory strategy during this unusual gait consists in a reduction of step length, although the reason of such a reduction is unclear. This is particularly puzzling because this smaller step length requires higher energy consumption, and is not an economical solution from the metabolic point of view (although it may be desirable for training and rehabilitation purposes (Zampeli et al., 2010)).

Subtle gait modifications were observed in case of ACL damage. It would be important to detect similar modifications in backward walking, since this task is more challenging and may unveil otherwise hidden imbalances.

Several explanations are possible for the improvement of proprioceptive sensibility in the ACL-R group, that is after tendon reconstruction, such as restoration of proprioceptive information from tendon stubs (Dhillon et al., 2010). It remains to be ascertained whether the same conclusion applies to unconscious proprioceptive information (spinocerebellar pathway), which has not been tested yet.

We did not observe any significant modification in step length during FW in subjects with the ACL lesion, what may be related to the high degree of automation of this motor sequence. However, subjects with ACL-D failed to reduce step length when performing BW. ACL surgical repair restores both proprioceptive sensibility and step length correction during BW.

This observation suggests that optimal proprioceptive information and/or mechanical stability from the knee are necessary to induce a reduction in step length during BW. Our hypothesis is that knee proprioception is an important signal used by the brain to reduce step length during the backward gait.

Several confounding factors should be taken into account: (i) ACL-D subjects significantly reduce their amount of physical activity and therefore their training status; (ii) ACL-R subjects suffer from quadriceps muscle hypotrophy and undergo extensive rehabilitation programs; (iii) it is impossible to disentangle the influence of the biomechanical stability from the proprioceptive loss. However, we did not find significant correlation between the amount of physical activity and step length during BW. Conversely, we observed a significant linear correlation between the knee proprioception threshold and step length during backward walking; this supports the main hypothesis that the modification of step size in BW requires a proprioceptive feedback from the knee.

We believe that these data offer interesting suggestions for some unsolved issues. Are gait modifications during backward walking simply the result of a biomechanical problem? Our data allow to conclude that there are situations in which the reduction of step size during backward walking is less evident. This phenomenon is difficult to explain purely on the biomechanical basis, because the gait parameters are not modified during forward walking. Finally, we also would like to underline the clinical relevance of backward walking parameters together with knee proprioceptive tests, to evaluate knee functionality before and after ACL reconstruction.

## Practical implications

Our observations suggest that the effects of the rehabilitation process can be evaluated by examining the number of steps during backward walking on a treadmill at 4–5km/h. Specifically, during backward walking, particularly at 5km/h, the number of steps should be much lower than in forward walking, if the rehabilitation procedure was successful.

## Figures and Tables

**Table 1:** IKDC and Tegner & Lysholm scales in the experimental groups (mean±S.D.)

Group	Tegner & Lysholm	IKDC
ACL-I (intact)	99.88±0.35	99.14±2.44
ACL-D (damaged)	68.86±18.18	64.41±15.45
ACL-R (repaired)	93.38±5.58	91.26±5.48
